# Alcohol Use Awareness in Pregnancy: A Perinatal Mental Health Audit

**DOI:** 10.1192/bjo.2026.11694

**Published:** 2026-06-30

**Authors:** Aliaa Ismail, Qurat Khurram, Alya Elmadih

**Affiliations:** South West community Perinatal Mental Health Team, Hampshire Isle of Wight Health Care NHS Foundation Trust, Hampshire, United Kingdom

## Abstract

**Aims:**

**Background**

There is no safe level of alcohol consumption during pregnancy, and NICE guidelines recommend complete abstinence. Even low levels of alcohol intake are associated with increased risks of miscarriage, prematurity, low birth weight, and Foetal Alcohol Spectrum Disorder. Women with mental health difficulties are at higher risk of alcohol use during pregnancy, potentially due to social disadvantage, increased stress, limited support networks, and reduced awareness of alcohol-related risks. Routine enquiry about alcohol use, including level and pattern of consumption, is therefore essential in all pregnant women.

**Aim**

To determine whether alcohol use is routinely discussed and documented during initial assessments of pregnant women referred to the South West community Perinatal Mental Health Team (SW-PMHT).

**Methods:**

A retrospective analysis of electronic records and assessment letters was conducted for all initial assessments of pregnant women referred to the SW-PMHT between April and June 2025. Of 48 identified patients, 13 were excluded due to discharge prior to assessment, non-attendance, or assessment by other services (e.g. CMHT or MBU). The final sample included 35 patients. Documentation was reviewed to determine whether alcohol use was explored during assessment.

**Results:**

Alcohol use was explored in 16 out of 35 patients (45.7%), with one patient disclosing alcohol consumption during pregnancy. Alcohol use was not explored in 19 patients (54.3%).

**Conclusion:**

This results demonstrated that alcohol use was not routinely explored in over half of pregnant women assessed by the SW-PMHT, representing a significant gap in care given the known risks of alcohol exposure during pregnancy. This highlights a clear need for improved and consistent screening practices.

**Actions and Re-audit**

Findings were presented at the multidisciplinary team meeting in September 2025, with action agreed to ensure routine enquiry and documentation of alcohol use at all initial assessments, using the AUDIT screening tool where appropriate.

**Completion of the cycle**

A re-audit was conducted using the same methodology over a three-month period (October–December 2025). Of 22 pregnant women included, alcohol use was explored in 17 patients (77.3%), compared to 5 patients (22.7%) where it was not explored, demonstrating significant improvement from previous results.

**Conclusion and future direction following completion of cycle**

The re-audit demonstrated improved compliance with assessment for alcohol use in pregnancy. To further enhance awareness and prevention, an information leaflet was developed and introduced for service users, outlining the risks of alcohol use during pregnancy and breastfeeding, with relevant resources and support links.

